# Phosphatidylserine Externalization in Cancer: Biology, Immune Suppression, and Emerging Theragnostic Strategies

**DOI:** 10.3390/ijms27020697

**Published:** 2026-01-09

**Authors:** Maro Yoo, Kyung-Hee Kim

**Affiliations:** 1 Center for Liver and Pancreatobiliary Cancer, National Cancer Center, Gyeonggi 10408, Republic of Korea; yoomaro@ncc.re.kr; 2 Biopharmaceutical Chemistry Major, Department of Applied Chemistry, School of Science and Technology, Kookmin University, Seoul 02707, Republic of Korea; 3 Antibody Research Institute, Kookmin University, Seoul 02707, Republic of Korea

**Keywords:** phosphatidylserine, cancer immunity, tumor microenvironment, PS-targeting therapy, immunosuppression, extracellular vesicles, theragnostics

## Abstract

Phosphatidylserine (PS) externalization is a conserved membrane stress signal that becomes chronically dysregulated in cancer cells and tumor-associated endothelium. In vivo, PS does not exist as a free lipid signal but is presented in specific membrane-associated forms, including apoptotic or stressed cell surfaces, PS-rich extracellular vesicles, and circulating lipid particles. Unlike apoptosis-associated transient PS exposure, malignant PS externalization arises from metabolic rewiring, oxidative stress, epigenetic silencing of flippases, and microenvironmental cues, creating an immunosuppressive interface across the tumor–host boundary. This review synthesizes mechanistic, immunological, and clinical evidence on PS biology, including its roles in tumor immune evasion, extracellular vesicle-mediated systemic suppression, and vascular remodeling. We further summarize the development and evaluation of PS-targeted therapeutic platforms—such as bavituximab, SapC-DOPS/BXQ-350, and PS-directed imaging agents—and highlight their translational potential in combination with radiotherapy, chemotherapy, and checkpoint inhibitors. Chronic PS externalization, as manifested through distinct cellular and vesicular carriers, represents a unifying biomarker of tumor stress, immune suppression, and therapeutic vulnerability, offering a next-generation axis for theragnostic cancer management.

## 1. Introduction

Phosphatidylserine (PS) is an anionic glycerophospholipid that does not function as a free signaling molecule in biological systems but instead exerts its effects through defined membrane-associated carriers [1,2]. Owing to its negative charge and amphipathic nature, PS is stably embedded within lipid bilayers and becomes extracellularly accessible only in specific structural contexts [2]. In vivo, PS is primarily encountered in three major forms: (i) externalized PS on the surface of cells undergoing apoptosis or non-apoptotic stress [3,4], (ii) PS-enriched extracellular vesicles released into blood and interstitial fluids [5], and (iii) PS-containing circulating lipid particles, such as plasma lipoproteins [2]. These distinct PS carriers differ in their biogenesis, spatial distribution, and biological consequences, collectively defining the functional landscape of PS biology in physiology and disease [1,2].

From a molecular perspective, PS recognition represents a nontrivial biochemical challenge requiring specialized sensing mechanisms [6,7]. PS is detected through two fundamentally distinct modes of interaction: direct molecular recognition by PS-binding proteins (e.g., annexins and related PS-binding domains) and electrostatic/biophysical recognition of PS-rich membrane surfaces as negatively charged platforms [3,6]. In the latter mode, proteins respond not to individual PS molecules but to collective membrane properties—most notably charge density and curvature—which can strongly shape binding and downstream activity [3,6].

This distinction is particularly critical in blood coagulation, where enzyme complex assembly depends on negatively charged phospholipid surfaces [8]. For example, coagulation reactions occurring on relatively flat PS-exposing cellular membranes can differ kinetically and structurally from those occurring on highly curved PS-rich vesicular surfaces [8,9]. In cancer, PS-exposing tumor-derived microparticles have been shown to provide highly efficient procoagulant platforms, thereby linking vesicle-associated PS to thrombin generation and downstream vascular consequences [9,10,11].

Importantly, PS externalization is not a binary on–off event but rather a dynamic and quantitatively regulated membrane phenomenon [3,4]. During classical apoptosis, PS becomes rapidly and transiently externalized following caspase-dependent disruption of lipid asymmetry [12,13], and this “eat-me” signal is a core component of apoptotic cell recognition and clearance [14,15]. Methodologically, widely used Annexin-V-based detection can preferentially report higher-density PS exposure and therefore may bias interpretation of the timing and extent of PS externalization in complex settings [16].

In contrast, accumulating evidence indicates that tumors often display persistent, non-apoptotic PS exposure on viable cancer cells and tumor-associated endothelium [17,18], as well as on circulating tumor-derived vesicles [5,19]. Such sustained PS exposure is increasingly recognized as a functional feature of tumor immune evasion and microenvironmental remodeling, rather than merely a terminal apoptotic hallmark [19,20,21]. Accordingly, PS biology in cancer should be interpreted within a framework that explicitly accounts for carrier context (cell vs. vesicle vs. particle) and methodological constraints in PS detection [16].

### 1.1. PS as an Active Immunoregulatory Molecule in the Tumor Microenvironment

PS externalization has broad immunological implications [15,20]. Surface PS engages phagocytic receptors and promotes tolerogenic efferocytosis programs [6,7], which can skew macrophages toward an M2-like phenotype and suppress pro-inflammatory cytokine production [20,22]. PS exposure also inhibits dendritic cell maturation and antigen-presenting capacity, thereby weakening T-cell priming [20,23].

In the vascular endothelium, stress-associated PS exposure on tumor blood vessels has been linked to pro-thrombotic remodeling and metastatic permissiveness [9,18]. Clinically and mechanistically, PS-exposing tumor-derived microparticles can amplify coagulation and promote tumor cell trans-endothelial migration [9,10]. In addition, tumor-derived PS-rich extracellular vesicles circulate systemically and reprogram distal immune niches; as shown in exosome biology and tumor-exosome immunology studies [5,24], these vesicles can impair effector T-cell function and support pre-metastatic niche formation (Table 1).

Collectively, PS functions as a broad-spectrum immunosuppressive ligand in the tumor microenvironment [20,21]. Unlike PD-L1/CTLA-4-centered suppression [25], PS simultaneously modulates multiple innate and adaptive pathways, making it a compelling therapeutic target.

### 1.2. Rationale for Targeting PS in Cancer Therapy

Given its accessibility on tumor surfaces and restricted exposure on normal tissues, PS has emerged as an attractive therapeutic target with broad immunoregulatory relevance [19,21,26]. Accordingly, multiple PS-targeting modalities—including monoclonal antibodies and vesicle-based platforms—have progressed into preclinical and clinical evaluation (Table 1).

Bavituximab binds PS indirectly via β_2_GP1 and has been investigated as an immune-modulating strategy in solid tumors [27,28], including clinical evaluation in NSCLC and other indications [27]. PS-targeted nanovesicles such as SapC-DOPS exploit saposin C’s affinity for PS-rich membranes; their therapeutic and diagnostic rationale has been detailed in glioblastoma-focused development work [29,30,31] and broader PS-targeting reviews [19,21] (Table 1).

Annexin-V-based probes and engineered PS-binding proteins have been widely used for PET/SPECT imaging of apoptosis and treatment response [16,32]. Newer PS-targeted nanoparticle tracers with optimized pharmacokinetics are under investigation for in vivo tumor imaging [33,34]. Strong synergy has been reported when PS-targeting approaches are combined with PD-1/PD-L1 blockade; this aligns with established checkpoint-therapy principles [25,35] and is frequently observed in PS-targeting combination strategies summarized in recent cancer-PS reviews [19,21,36] (Table 1).

### 1.3. Clinical Implications and Unmet Needs

Despite robust preclinical activity, PS-targeting therapeutics have shown mixed clinical outcomes. For example, bavituximab demonstrated manageable toxicity but failed to meet the primary endpoint in a Phase III NSCLC trial [27], highlighting the need for improved patient selection strategies emphasized in the broader PS-targeting field [19,28,37].

Mechanistically, existing immunotherapies often address adaptive resistance pathways [38], whereas PS-targeting agents modulate myeloid-driven innate immune suppression and efferocytosis-linked tolerance programs [20,21,22], potentially expanding therapeutic benefit across “cold” tumor settings. Additionally, PS-exposing tumor-derived vesicles represent a systemic immunosuppressive axis that is not directly addressed by checkpoint inhibitors alone [5,24,39] (Table 1).

### 1.4. Aim of This Review

This review integrates mechanistic and translational evidence regarding PS externalization and its roles in tumor progression and immune modulation, including foundational membrane biology [1,2,4] and cancer-focused PS targeting frameworks [19,21]. We summarize biochemical regulation of PS exposure, describe tumor-associated PS functions, review therapeutic platforms, and highlight emerging diagnostic applications [16,21,33]. The strengths, limitations, and future perspectives of PS-targeting oncology are discussed, with emphasis on receptor biology [6,7,40] and clinical translation considerations [27,28,37]. Our objective is to provide a comprehensive framework for PS as a therapeutic and diagnostic target in modern oncology (Table 1).

## 2. Biology and Mechanisms of Phosphatidylserine Exposure

**Figure 1:**
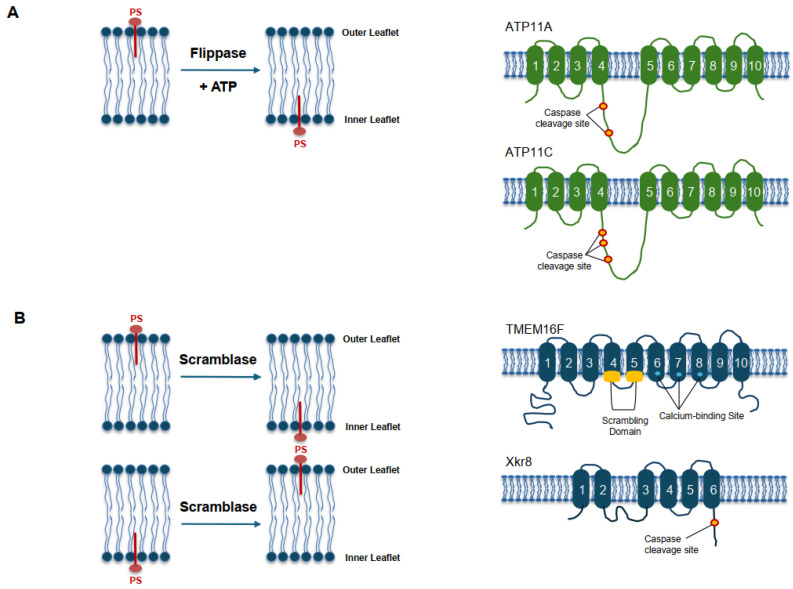
Maintenance and disruption of phosphatidylserine (PS) asymmetry in the plasma membrane. (**A**) Homeostatic maintenance of PS asymmetry in resting cells. Under physiological conditions, phosphatidylserine (PS) is selectively confined to the inner leaflet of the plasma membrane. This asymmetric distribution is actively maintained by ATP-dependent P4-ATPase flippases, primarily ATP11A and ATP11C, which continuously transport PS from the outer to the inner leaflet. In contrast, phospholipid scramblases are present but remain functionally inactive under basal conditions, thereby preserving membrane asymmetry and preventing extracellular PS exposure. (**B**) Loss of PS asymmetry following cellular stress or apoptotic signaling. Upon apoptotic stimulation or strong cellular stress, membrane asymmetry collapses as flippase activity is suppressed and scramblases become activated. Caspase-dependent cleavage or Ca^2^⁺-mediated activation of scramblases enables bidirectional phospholipid movement across the bilayer, resulting in rapid externalization of PS on the outer leaflet. This transition converts PS from a cryptic inner-leaflet lipid into a surface-exposed biological signal that mediates downstream immune, coagulation, and clearance responses.

PS asymmetry is dynamically regulated by three major classes of membrane lipid transporters—flippases, floppases, and scramblases—which collectively determine the distribution of aminophospholipids across the plasma membrane bilayer (Figure 1) [1,4,41]. In healthy cells, ATP-dependent P4-ATPase flippases such as ATP11A and ATP11C actively maintain PS on the inner leaflet [12,41]. Upon apoptotic or strong activation signals, caspase-mediated cleavage of flippases or Ca^2+^ influx disrupts membrane asymmetry and activates scramblases, including TMEM16F and Xkr8, leading to rapid PS externalization [42,43,44]. In cancer, this regulatory balance is frequently altered by oxidative stress and metabolic rewiring, resulting in sustained PS exposure on viable cells [17,18,19] (Table 2).

### 2.1. Enzymatic Regulation of PS Asymmetry

Under basal conditions, PS is retained on the cytoplasmic leaflet by P4-ATPase flippases, which consume ATP to counteract passive outward diffusion [12,41]. ATP11A and ATP11C represent the dominant flippases in epithelial, hematopoietic, and endothelial cells [12], and their function depends on the β-subunit CDC50A for proper folding and trafficking [12].

During apoptosis, caspase activation cleaves ATP11A/ATP11C, thereby disabling inward PS transport [12]. Concurrently, scramblases such as Xkr8—activated by caspase cleavage [45]—and TMEM16F—activated by Ca^2+^ influx [42]—facilitate bidirectional phospholipid movement and collapse membrane asymmetry [4]. In cancer cells, reduced flippase expression together with persistent TMEM16F activity contributes to chronic PS externalization even in the absence of apoptosis [17,19] (Table 1).

### 2.2. Role of Ca^2+^ Signaling and Scramblase Activation

Cytosolic Ca^2+^ elevation is a major driver of PS scrambling. TMEM16F (ANO6) responds directly to increased intracellular Ca^2+^, mediating non-selective trans-bilayer phospholipid movement [42]. Cancer cells frequently exhibit dysregulated Ca^2+^ homeostasis due to ER stress and mitochondrial dysfunction, enabling tonic TMEM16F activation and sustained PS exposure [46].

By contrast, Xkr8 activation is primarily caspase-dependent and classically associated with apoptosis [45,47,48]. However, inflammatory and cytotoxic conditions within the tumor microenvironment can induce sublethal caspase activity, resulting in partial Xkr8 activation and incomplete yet persistent PS exposure [19,46] (Table 1).

### 2.3. Oxidative Stress, ROS Accumulation, and Mitochondrial Dysfunction

Cancer cells generate elevated levels of reactive oxygen species (ROS) as a consequence of oncogenic signaling and mitochondrial stress [49,50]. Oxidative damage to membrane lipids disrupts flippase activity while favoring scramblase-mediated lipid redistribution [49]. In particular, peroxidation of polyunsaturated PS species destabilizes inner leaflet retention and promotes outward drift [49,50].

**Figure 2:**
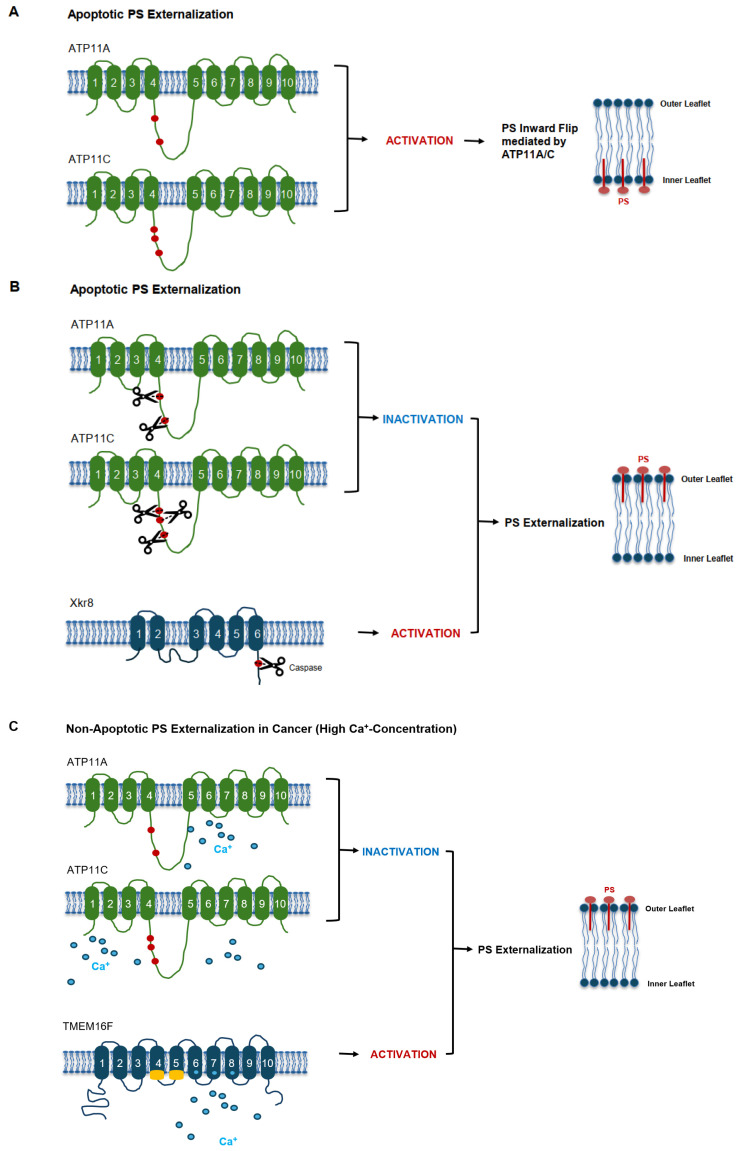
Mechanistic spectrum of phosphatidylserine externalization: homeostatic maintenance, apoptotic exposure, and non-apoptotic stress induction. (**A**) Apoptotic PS externalization mediated by caspase-dependent flippase inactivation and scramblase activation. During apoptosis, caspase cleavage inactivates the flippases ATP11A and ATP11C, abolishing their inward PS transport. Concurrently, caspase-dependent activation of the scramblase Xkr8 triggers rapid, bidirectional phospholipid scrambling that results in stable PS exposure on the outer leaflet. This apoptotic PS externalization serves as an “eat-me” signal for phagocytes. (**B**) Caspase-dependent activation of Xkr8 drives apoptotic scrambling and sustained PS exposure. Apoptotic signaling activates Xkr8 by caspase-mediated cleavage, enabling high-efficiency scrambling of phospholipids and the outward exposure of PS. Simultaneous loss of ATP11A/ATP11C activity consolidates PS externalization. Together, these events generate a persistent apoptotic PS signal recognized by TIM-4, Stabilin-2, and other PS receptors on phagocytes. (**C**) Non-apoptotic PS externalization induced by elevated intracellular Ca^2+^ in cancer and stressed cells. Non-apoptotic PS exposure arises from Ca^2+^-dependent activation of TMEM16F scramblase and inactivation of ATP11C, without caspase involvement. Tumor cells subjected to hypoxia, ROS accumulation, metabolic stress, or high intracellular Ca^2+^ display chronic, non-apoptotic PS exposure. This persistent signal differs from apoptotic PS by retaining cell viability and contributing to immune evasion, extracellular vesicle (EV) release, and tumor–host interaction.

ROS can additionally impair ATP11A/ATP11C through oxidation of critical thiol residues, further suppressing inward PS transport [50]. Mitochondrial permeability transition, common in metabolically stressed tumor cells, increases cytosolic Ca^2+^ and amplifies TMEM16F-driven PS externalization [46,50] (Figure 2).

### 2.4. Metabolic Rewiring and ATP Depletion

Altered cancer metabolism directly influences PS asymmetry by modulating ATP availability. Because flippases are ATP-dependent, metabolic stress preferentially suppresses inward PS transport while leaving Ca^2+^-driven scrambling relatively intact [46,49,50]. Hypoxia and nutrient deprivation—hallmarks of the tumor microenvironment—therefore bias membranes toward sustained PS exposure [49].

Acidic extracellular pH and lactate accumulation further alter membrane biophysics and reduce the energetic barrier for PS externalization [49,50]. In multiple tumor types, ATP11A and ATP11C expression is transcriptionally or epigenetically suppressed, reinforcing this imbalance [12,49] as summarized in Table 2.

### 2.5. Inflammation-Driven and Cytokine-Mediated PS Exposure

Inflammatory cytokines within the tumor microenvironment strongly influence membrane dynamics. TNF-α enhances ceramide production and cytoskeletal remodeling [50], while IFN-γ promotes mitochondrial ROS generation and Ca^2+^ flux [46,51]. IL-1β further stimulates Ca^2+^-dependent phospholipid scrambling [44,52]. Together, these signals induce PS exposure on cancer cells and tumor-associated endothelial cells without triggering apoptosis [17,18,46] (Table 2).

During angiogenesis, endothelial activation driven by VEGF and inflammatory mediators also promotes PS externalization, facilitating coagulation and leukocyte adhesion within tumor vasculature [8,10,18] (Table 1).

### 2.6. PS Exposure During Extracellular Vesicle (EV) Biogenesis

Extracellular vesicles (EVs), including microvesicles and exosomes, are intrinsically enriched in PS as a consequence of membrane curvature and budding mechanics [5,53]. Microvesicle shedding involves cytoskeletal relaxation and outward membrane curvature, conditions that favor PS externalization [5,53]. In cancer, elevated Ca^2+^ signaling and TMEM16F activation further promote release of PS-rich microvesicles [44,53].

**Figure 3:**
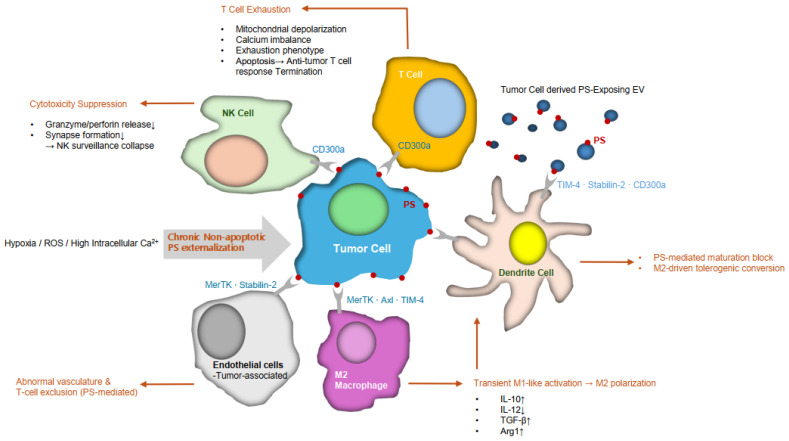
PS-driven immunosuppressive network in the tumor microenvironment (TME). Chronic, non-apoptotic PS externalization from viable tumor cells—driven by hypoxia, ROS, and elevated intracellular Ca^2+^—initiates a multi-layered immunosuppressive cascade. Tumor cells and PS-exposing extracellular vesicles (EVs) engage PS receptors (MerTK, Axl, TIM-4) on macrophages, inducing a transient M1-like activation followed by M2 polarization characterized by IL-10↑, IL-12↓, TGF-β↑, Arg1↑. M2 macrophage-derived cytokines together with direct PS/EV signaling suppress dendritic cells via PS-mediated maturation block and M2-driven tolerogenic conversion, marked by TIM-4, Stabilin-2, and CD300a engagement. Consequently, T cells encountering chronic PS signaling through CD300a develop mitochondrial depolarization, Ca^2+^ imbalance, exhaustion, and apoptosis, resulting in the collapse of antitumor immunity. NK cells undergo granzyme/perforin suppression and impaired synapse formation through PS–CD300a signaling, compromising innate cytotoxic surveillance. Tumor-associated endothelial cells expressing MerTK and Stabilin-2 internalize PS^+^ EVs and undergo PS-mediated vascular remodeling, generating abnormal vasculature and T-cell exclusion that reinforce immune evasion. Together, these pathways define a PS-centric immunoregulatory network that shapes the suppressive tumor microenvironment.

Once released, PS-positive EVs circulate systemically and exert immunosuppressive effects, including macrophage M2 polarization and suppression of dendritic cell maturation [5,24], as well as functional impairment of effector T cells [5,24,39]. These processes contribute to pre-metastatic niche formation and endothelial modulation, as summarized in Table 2 and illustrated in Figure 2 and Figure 3.

## 3. Roles of PS in Tumor Immunology and Tumor Progression

### 3.1. PS as an Immunoregulatory “Checkpoint-like” Signal in the Tumor Microenvironment

Although PS is classically described as an apoptotic “eat-me” signal [3,14,15], cancer cells frequently display persistent PS exposure on viable tumor cells, tumor vasculature, and tumor-derived extracellular vesicles [5]. Unlike PD-L1 or CTLA-4 ligands, which suppress T-cell signaling through discrete receptor pathways [25], PS engages multiple innate and adaptive immune receptors, broadly reprogramming the tumor microenvironment toward immune tolerance [20,21].

PS-binding receptors—including TIM-4, Stabilin-2, BAI1, CD300a, and TAM family kinases such as MerTK and Axl [5,20]—initiate anti-inflammatory signaling cascades upon ligation [20,54]. These cascades suppress dendritic cell activation and antigen presentation while promoting macrophage immune-regulatory phenotypes [20,22], ultimately dampening effector T-cell recruitment and function [20,21,24]. As a result, PS operates as a multi-axis immune checkpoint coordinating suppression across both innate and adaptive immune branches (Figure 3).

Importantly, PS exposure is further increased following chemotherapy, radiotherapy, or anti-angiogenic treatment, reinforcing its role as a stress-induced immunomodulator rather than a passive marker of cell death [19,21,55].

### 3.2. Impact of PS on Macrophage Polarization and Myeloid Immunity

#### 3.2.1. M2-like Skewing via TAM and TIM Family Receptors

Tumor-associated macrophages (TAMs) are among the immune populations most profoundly affected by PS. Chronic exposure to PS-rich tumor membranes or extracellular vesicles skews macrophages toward an M2-like phenotype characterized by reduced pro-inflammatory cytokine production and enhanced tissue remodeling [20,22,24]. This shift is accompanied by increased IL-10, TGF-β, and arginase-1 expression and impaired antigen-presenting capacity [22,56].

Mechanistically, PS ligation activates TAM receptors such as MerTK and Axl, initiating PI3K/Akt signaling while suppressing NF-κB activation [40,54]. Although this pathway is essential for physiological efferocytosis [15,30], tumors co-opt it to maintain an immunosuppressive niche (Table 1 and Table 2; Figure 3).

#### 3.2.2. Suppression of Dendritic Cell Maturation

Exposure to PS-expressing tumor cells or PS-containing vesicles inhibits dendritic cell maturation and antigen presentation [5] (Table 1).

### 3.3. PS Modulation of T-Cell Immunity

#### 3.3.1. Direct Inhibition of T-Cell Activation

PS-rich extracellular vesicles can directly induce apoptosis or functional dysfunction in activated T cells [5,24,39]. Engagement of PS-recognizing inhibitory receptors promotes mitochondrial depolarization, ROS accumulation, and impaired TCR signaling, resulting in reduced IFN-γ production [24]. Tumors with high PS exposure therefore exhibit reduced densities of tumor-infiltrating lymphocytes and diminished cytotoxic activity [17,18,19] (Table 1).

#### 3.3.2. Indirect Suppression Through Myeloid Regulation

In addition to direct effects, PS indirectly suppresses T-cell immunity through dysregulation of macrophage and DC function. Impaired antigen presentation and cross-priming result in defective memory T-cell formation [35], a mechanism distinct from checkpoint ligands such as PD-L1, which primarily act on pre-existing T-cell responses [25]. Thus, PS limits anti-tumor immunity predominantly at the level of immune initiation [20,23] (Table 1).

### 3.4. PS-Mediated Remodeling of Tumor-Associated Vasculature

#### 3.4.1. Endothelial PS Exposure as a Tumor-Specific Phenomenon

PS exposure on endothelial cells represents a defining feature of tumor vasculature [18]. Chronic oxidative stress, VEGF-driven permeability, disordered shear stress, and Ca^2+^ dysregulation collectively disrupt membrane asymmetry in tumor endothelium [6,14], resulting in luminal PS exposure (Table 2).

#### 3.4.2. Consequences for Angiogenesis and Immune Infiltration

PS-exposed endothelial cells contribute to local thrombogenesis [8,9], abnormal angiogenesis, impaired leukocyte trafficking, and enhanced metastatic adhesion [10,11,56,57]. These vascular changes create both physical and biochemical barriers to immune infiltration, reinforcing immune exclusion within tumors [18,33] (Table 1 and Table 2).

### 3.5. PS-Positive Extracellular Vesicles and Systemic Immunosuppression

#### 3.5.1. Composition and Release of PS-Exposing EVs

Tumor cells and stressed stromal cells release PS-rich extracellular vesicles, including exosomes and microvesicles, enriched in immunomodulatory proteins and pro-coagulant factors [5,9]. Their release is enhanced by hypoxia, radiation, and chemotherapy, making them abundant in advanced cancer [5,19,55] (Table 1).

#### 3.5.2. Effects on Systemic Immunity

Circulating PS-positive EVs promote macrophage M2 polarization [5,24,39], suppress dendritic cell maturation [5,24], and induce functional exhaustion of activated T cells [5,24,39]. These effects extend beyond the primary tumor, facilitating pre-metastatic niche formation and systemic immune suppression (Table 1).

### 3.6. Role of PS in Coagulation, Thrombosis, and Metastatic Spread

Aberrant PS exposure on tumor cells provides a negatively charged scaffold for coagulation enzyme assembly, thereby promoting a hypercoagulable phenotype [8,10]. Annexin V inhibition studies demonstrated that tumor-associated procoagulant activity depends on surface-accessible PS rather than total PS abundance [10,11].

Tumor cells co-expressing tissue factor and PS efficiently assemble intrinsic tenase and prothrombinase complexes, resulting in robust thrombin generation [8,10,11]. PS-positive tumor-derived microvesicles further amplify systemic thrombotic risk by serving as highly efficient catalytic platforms [9,11]. These mechanisms were established in landmark melanoma and glioma studies [10,11].

### 3.7. PS-Dependent Metastatic Fitness Beyond Coagulation

PS exposure contributes to metastatic dissemination through mechanisms that extend beyond coagulation alone. PS-positive tumor cells exhibit enhanced platelet interaction and vascular adhesion [50,57], facilitating arrest within the microvasculature. Importantly, PS-dependent procoagulant activity has been demonstrated on viable, non-apoptotic tumor cells [10,17], indicating active membrane remodeling rather than terminal cell death.

PS also promotes immune evasion during circulation by engaging PS-recognizing receptors on macrophages and endothelial cells, suppressing pro-inflammatory signaling and immune clearance [20,53]. Thus, PS functions as a multifunctional membrane signal integrating coagulation-dependent and immune-regulatory cues. Experimental evidence supports PS-dependent metastatic fitness independent of overt thrombosis [50,57], while PS-containing liposomes targeting vascular adhesion molecules further highlight the vascular specificity of PS-mediated interactions [33].

### 3.8. Chronic PS Exposure as a Hallmark of Tumor Stress Biology

Chronic PS exposure correlates with ER stress, mitochondrial dysfunction, oxidative injury, nutrient deprivation, and defective autophagy in cancer cells [46,49,51]. This persistent exposure represents a form of stress-associated membrane reprogramming that distinguishes tumor cells from healthy tissues [17,18,19]. Notably, PS remains exposed in the absence of apoptotic markers, reinforcing its utility as a selective therapeutic and diagnostic target [19,21] (Figure 2).

## 4. Therapeutic Targeting of Phosphatidylserine in Cancer

**Figure 4:**
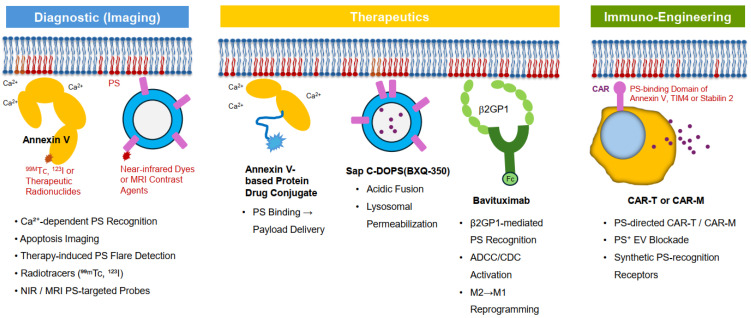
Phosphatidylserine-targeted diagnostic, therapeutic, and immuno-engineering platforms. Tumor cells, stressed stromal cells, and tumor-associated endothelial cells aberrantly externalize phosphatidylserine (PS), creating a shared vulnerability across multiple therapeutic modalities. In this schematic, colors are used to distinguish functional modules rather than quantitative differences. Blue denotes diagnostic and imaging platforms, yellow represents direct therapeutic interventions targeting PS-exposing membranes, and green indicates immuno-engineering and immune-modulatory strategies. Gray shading is used for background cellular and stromal components. (Left) Diagnostic imaging platforms: Ca^2+^-dependent Annexin V and its radiolabeled derivatives (^99^ᵐTc, ^123^I) enable visualization of apoptosis, early treatment response, and therapy-induced PS flare. Near-infrared and MRI-based PS probes allow noninvasive tumor detection independent of caspase activity. (Center) Therapeutic platforms: Bavituximab binds PS via β_2_-glycoprotein 1, promoting Fc-mediated effector functions (ADCC/CDC) and M2-to-M1 macrophage reprogramming within the immunosuppressive tumor microenvironment. The fusogenic nanovesicle SapC-DOPS (BXQ-350) targets acidic PS-rich membranes to induce lysosomal permeabilization and caspase-independent cell death. Annexin-V-based protein–drug conjugates use selective PS affinity to deliver cytotoxic payloads directly to PS-positive tumor membranes. (Right) Immuno-engineering platforms: Next-generation strategies, including PS-directed CAR-T and CAR-macrophage constructs, synthetic PS-recognition domains, and PS^+^ extracellular-vesicle blockade, aim to overcome PS-driven immune suppression, restore phagocyte activation, and enhance antigen presentation. Collectively, these PS-targeted modalities provide a unified framework linking tumor imaging, direct tumor clearance, and immune reprogramming.

Chronic externalization of PS on tumor cells, stromal cells, and tumor-associated vasculature provides a unique opportunity for therapeutic targeting [17,18,19,21]. Unlike classical immune checkpoints that primarily regulate T-cell activation [25], PS-driven immunosuppression integrates innate, adaptive, and stromal signals [20,21]. Therapeutic platforms targeting PS include monoclonal antibodies, nanovesicles, engineered PS-binding proteins, immunomodulatory adjuvants, and emerging cellular therapies (Table 1) (Figure 4) [58]. PS-targeted nanoparticles leveraging electrostatic recognition of PS-rich membranes have also been developed as delivery platforms, enabling selective tumor accumulation and therapeutic payload delivery [58]. These strategies differ in mechanism, pharmacokinetics, antigen density requirements, and translational maturity.

### 4.1. Monoclonal Antibodies Targeting PS

#### 4.1.1. Mechanism: β_2_GP1-Mediated Bridging and Immune Activation

Bavituximab is the most clinically advanced PS-targeting monoclonal antibody [27,28]. Rather than binding PS directly, bavituximab binds β_2_-glycoprotein I (β_2_GP1), which associates with exposed PS on tumor cells and stressed endothelial cells [27,28]. This PS–β_2_GP1 complex enables FcγR cross-linking and antibody-dependent cellular cytotoxicity, contributing to remodeling of the immunosuppressive tumor microenvironment [27,28].

Upon binding, bavituximab can repolarize tumor-associated macrophages toward pro-inflammatory phenotypes and promote dendritic cell maturation and antigen presentation [27,28] (Table 3). These effects support improved NK- and T-cell-mediated anti-tumor responses [27,28].

#### 4.1.2. Preclinical Efficacy

Preclinical studies show robust activity of PS-targeting antibodies in multiple tumor models, including lung cancer, hepatocellular carcinoma, melanoma, and brain tumors [19,21,27,60]. In murine systems, PS-targeting antibodies synergize with PD-1/PD-L1 or CTLA-4 blockade by reversing myeloid suppression and enhancing antigen presentation [20,25,36]. Combinations with radiotherapy or chemotherapy can further enhance efficacy, in part because cytotoxic therapies increase PS exposure on tumor vasculature and cancer cells [19,55] (Table 1).

#### 4.1.3. Clinical Evaluation of Bavituximab

Phase I studies suggested an acceptable safety profile for bavituximab, with manageable infusion reactions and immune-related adverse events [27,28]. In early-phase studies in NSCLC and hepatocellular carcinoma, bavituximab plus chemotherapy showed encouraging response signals compared with historical controls [28,59]. However, the Phase III SUNRISE trial did not meet its primary endpoint, likely reflecting patient heterogeneity and the lack of biomarker-based stratification [27,28,37].

Secondary analyses suggest that benefit may be enriched in PS-high tumors or immune-inflamed microenvironments, particularly in settings where therapy increases PS exposure [19,54] (Table 3).

### 4.2. Engineered PS-Binding Proteins

#### 4.2.1. Annexin V and Its Derivatives

Annexin V binds PS with high affinity in a Ca^2+^-dependent manner [16]. Although widely used as an apoptosis tracer [16,32], engineered Annexin V variants have been developed for diagnostic and theranostic applications, including multimeric formats and labeled imaging probes [16,32]. However, Ca^2+^ dependency and rapid clearance can limit in vivo performance and may bias detection toward higher-density PS exposure (Table 1).

#### 4.2.2. Other PS-Binding Domains

Other PS-binding modalities—including lactadherin (MFG-E8) and engineered PS-binding peptides—have been explored as targeting modules for imaging and delivery platforms [34,56,60]. Engineered PS-binding peptides constitute a distinct class of ligands with advantages in small size and modular design and have been applied to PS-directed targeting and delivery strategies [60]. These platforms exploit distinct biochemical properties such as Ca^2+^-independent binding (lactadherin) and recognition of PS-rich membrane microdomains (Table 1).

### 4.3. SapC–DOPS and BXQ-350: PS-Targeting Nanovesicles

#### 4.3.1. Mechanism: Saposin C–Driven Lysosomal Fusion

SapC–DOPS nanovesicles consist of saposin C combined with dioleoylphosphatidylserine (DOPS) [29,30,31]. Saposin C preferentially interacts with PS-rich membranes under acidic conditions, enabling selective fusion with tumor cells that exhibit surface acidity and abundant PS exposure [29,30,31]. This fusion promotes intracellular delivery and can trigger apoptotic or necrotic tumor cell death [30,31].

Because normal cells generally maintain PS on the inner leaflet and lack acidic extracellular microenvironments, SapC–DOPS demonstrates tumor selectivity [29,30] (Table 2).

#### 4.3.2. Preclinical and Clinical Evaluation

SapC–DOPS has shown activity in glioblastoma and other solid tumor models, inducing tumor regression and prolonging survival in preclinical studies [30,31,55,61]. BXQ-350, a clinical-grade derivative, demonstrated acceptable safety in Phase I studies, with ongoing clinical evaluation across tumor types [29,31]. Its proposed therapeutic index derives from combined sensitivity to tumor acidity and PS exposure, which are accentuated under microenvironmental stress conditions [29,30,31] (Table 1).

### 4.4. Combination Strategies Involving PS Targeting

#### 4.4.1. Synergy with Immune Checkpoint Inhibitors

PS-targeted agents can promote antigen presentation and dendritic cell maturation while reversing myeloid immunosuppression [20,27,36], complementing PD-1/PD-L1 or CTLA-4 blockade [25,35]. Multiple preclinical studies report improved tumor control when PS-targeting antibodies or nanovesicles are combined with checkpoint inhibitors [21,25,36] (Table 1).

#### 4.4.2. Combination with Radiotherapy and Chemotherapy

Radiotherapy increases PS exposure through ROS generation and membrane damage, thereby increasing accessibility of PS-targeted agents [49,54]. Chemotherapeutic agents can similarly elevate PS exposure as part of treatment-induced stress responses [19,55,62]. These dynamics support consistent preclinical synergy between PS targeting and cytotoxic modalities [55,62] (Table 1).

#### 4.4.3. Theranostic Applications

PS is a promising theranostic biomarker because it reflects tumor stress, immune remodeling, and treatment-induced membrane perturbation [16,19,21]. PS-directed imaging agents—including Annexin V derivatives and labeled antibodies—enable visualization of apoptosis and treatment response [16,32]. When paired with PS-targeted therapeutic platforms, imaging can support real-time monitoring and response-adaptive treatment strategies [16,32,33] (Table 1).

### 4.5. Limitations and Challenges of PS-Targeted Therapy

Despite substantial promise, PS-targeting strategies face key translational challenges. PS exposure is heterogeneous across tumor types and treatment states, complicating uniform targeting strategies [19,27,37]. Many PS-binding modalities also suffer from pharmacokinetic limitations, including rapid clearance, which may necessitate engineering for improved stability and systemic exposure [16,63]. Off-target binding to PS on activated platelets, inflammatory cells, and apoptotic debris can reduce tumor specificity [8,16], while manufacturing and stability constraints remain significant for nanovesicle and fusion-protein platforms [29,31]. Finally, the lack of validated biomarkers to define PS-high tumors limits patient stratification and precision clinical trial design [27,37].

Addressing these barriers will be essential for improving clinical translation and identifying patient subsets most likely to benefit (Table 2) (Figure 4).

## 5. Clinical Applications and Translational Potential of PS-Targeted Platforms

PS externalization represents an accessible and broadly expressed biomarker across multiple tumor types and disease stages [17,18,19]. Because PS exposure increases in response to chemotherapy, radiotherapy, hypoxia, and oxidative stress, PS-targeting agents can exploit stress-induced vulnerabilities and complement existing treatment modalities [19,50,54]. Accordingly, clinical applications of PS-targeting platforms fall into three major categories: direct anti-tumor therapy, immunomodulation and combination strategies, and PS-based imaging and theranostics (Table 1).

### 5.1. Direct Therapeutic Applications

#### 5.1.1. Single-Agent Activity and Tumor Targeting

Several PS-directed modalities, including bavituximab and SapC–DOPS/BXQ-350, have demonstrated anti-tumor activity in preclinical models and early-phase clinical trials [27,29,31]. PS-targeting antibodies mediate antibody-dependent cellular cytotoxicity and enhance macrophage-mediated phagocytosis, thereby alleviating M2-dominant myeloid suppression [27,28]. In parallel, PS-targeted nanovesicles such as SapC–DOPS selectively fuse with PS-rich tumor membranes, enabling intracellular delivery of cytotoxic or lysosomal mediators that induce tumor cell death [29,30,31].

These effects are most pronounced in tumors with high basal PS exposure or in settings where conventional therapies increase PS availability [17,19,55] (Table 2).

#### 5.1.2. Targeting Tumor Vasculature

PS exposure on tumor-associated endothelial cells provides a rationale for vascular-targeted therapy [18,33]. PS-targeting antibodies preferentially accumulate within abnormal tumor vasculature and initiate Fc-dependent immune activation, thereby reversing vascular immunosuppression and improving leukocyte trafficking [27,33]. In preclinical models, PS-directed therapies normalize tumor vasculature, improve oxygenation, and sensitize tumors to radiotherapy and chemotherapy [33,55] (Table 1).

#### 5.1.3. Targeting PS-Positive Extracellular Vesicles

PS-positive extracellular vesicles constitute a systemic immunosuppressive axis that contributes to metastatic progression and myeloid reprogramming [5,24,39]. PS-targeting antibodies and engineered PS-binding proteins can neutralize circulating EVs, restoring anti-tumor immune responses [24,39]. Unlike checkpoint blockade, which primarily acts on tumor-infiltrating T cells [25], EV-directed PS targeting extends immunomodulation beyond the primary tumor site (Table 1).

#### 5.1.4. Pharmacokinetic and Delivery Limitations

Many PS-binding proteins, including Annexin V, exhibit rapid clearance and short plasma half-lives, limiting systemic exposure [16]. Nanovesicle- and fusion-protein-based platforms require optimized formulation to maintain stability, cargo retention, and PS-binding affinity [29,31,63]. These limitations highlight the need for advanced engineering approaches, including Fc fusion and PEGylation, to improve pharmacokinetics and delivery efficiency (Table 1).

### 5.2. Combination Therapy and Immunomodulatory Applications

#### 5.2.1. Synergy with Checkpoint Inhibitors

PS-targeting agents complement PD-1/PD-L1 and CTLA-4 blockade by reversing myeloid-driven immunosuppression and enhancing antigen presentation [20,25,27,36]. In multiple preclinical models, PS-targeting antibodies promote dendritic cell maturation and TAM repolarization, resulting in improved tumor control when combined with checkpoint inhibitors [25,36] (Table 1).

#### 5.2.2. Combination with Radiotherapy

Radiotherapy induces acute PS exposure through ROS generation, membrane oxidation, and scramblase activation [49,55]. PS-targeted agents exploit this induced vulnerability, producing synergistic anti-tumor effects in several tumor models [55]. Combined treatment enhances tumor regression, delays recurrence, and promotes immunogenic cell death [35,55] (Table 1).

#### 5.2.3. Combination with Chemotherapy

Chemotherapeutic agents such as paclitaxel and doxorubicin increase PS exposure as part of cellular stress responses [35,61]. This chemotherapy-induced PS exposure enhances antibody binding, ADCC, and nanovesicle-mediated payload delivery [62], providing a mechanistic basis for combination strategies (Table 1).

### 5.3. PS-Targeted Imaging and Theranostics

#### 5.3.1. PS Imaging for Apoptosis and Treatment Monitoring

PS has long served as a biomarker for apoptosis, with Annexin-V-based PET and SPECT tracers enabling visualization of treatment-induced cell death [16,32]. Beyond apoptosis imaging, engineered PS-targeting probes can detect tumor burden and radiation-induced membrane stress [16,33], offering dynamic insights into therapeutic response (Table 1).

#### 5.3.2. Theranostic Fusion Platforms

Theranostic platforms combining PS targeting with imaging or therapeutic payloads are emerging as next-generation tools in oncology [19,21,33]. Radiolabeled PS-binding antibodies and PS-directed nanoparticles permit simultaneous tumor imaging and targeted cytotoxicity [16,32,33], enabling precision-guided therapy selection and response monitoring (Table 1).

### 5.4. Future Directions and Clinical Development Needs

Despite promising translational progress, several challenges remain. PS exposure varies across tumor types and treatment states [19,27], complicating uniform targeting strategies. Pharmacokinetic limitations, including rapid clearance of PS-binding proteins, and off-target binding to PS on activated platelets or apoptotic debris may reduce specificity [16,63]. In addition, nanovesicle manufacturing and stability constraints persist [29,31,61], and the lack of validated biomarkers for PS-high tumors limits precision trial design [27,37]. Addressing these unmet needs will require advances in biomarker development, delivery optimization, and mechanistic understanding of stress-induced PS exposure (Table 1).

## 6. Conclusions

The therapeutic relevance of PS arises not only from its unique exposure on viable tumor cells and tumor-associated endothelium but also from its sensitivity to treatment-induced stress. Chemotherapy, radiotherapy, anti-angiogenic therapy, and metabolic perturbations all increase PS exposure, thereby enhancing the accessibility of PS-targeting agents [19,49,55]. These stress-dependent dynamics position PS as both a therapeutic vulnerability and a dynamic biomarker capable of monitoring treatment response, tumor burden, and immunologic remodeling (Table 1).

Multiple classes of PS-targeted platforms—including monoclonal antibodies, nanovesicles, imaging agents, and engineered PS-binding proteins—have demonstrated efficacy in preclinical models and early-phase clinical trials [19,27]. Their ability to repolarize myeloid cells, enhance antigen presentation, normalize tumor vasculature, and reduce systemic immunosuppression highlights their complementary role alongside checkpoint inhibitors and conventional cytotoxic therapies [20,25]. Nevertheless, several obstacles remain, including heterogeneous PS expression, rapid clearance of certain PS-binding proteins, off-target interactions, formulation complexity, and the absence of validated biomarkers for identifying PS-high tumors [16,37].

Advancing PS-directed oncology will require integrated efforts across mechanistic biology, therapeutic engineering, and clinical development (Supplementary Figure S1). Key priorities include establishing robust biomarkers for PS-high tumors; optimizing pharmacokinetic properties through molecular engineering; improving manufacturing and stability of nanovesicles and fusion proteins; and designing combination regimens informed by the kinetics of treatment-induced PS exposure. With these developments, PS has the potential to evolve from a membrane stress signal into a central axis of next-generation cancer theranostics—one that links molecular stress biology, immune reprogramming, and precision-guided treatment strategies.

## Figures and Tables

**Table 1:** PS-Targeted Imaging and Translational Challenges.

Application	PS Carrier Detected	Imaging Modality	Experimental Model	Limitation	Key Reference
Apoptosis imaging	Apoptotic cell surface PS	Annexin V–SPECT/PET	Cancer patients	Low specificity for non-apoptotic PS	[7]
Therapy response monitoring	Tumor cell + EV PS	Molecular imaging probes	Mouse tumor models	Signal overlap with inflammation	[7]
Vascular PS imaging	Endothelial PS	Targeted contrast agents	Preclinical vasculature models	Translation barrier	[14]

Notes: This table highlights unresolved technical and biological challenges in PS-based imaging strategies.

**Table 2:** Modes of Phosphatidylserine Exposure and Functional Consequences in Cancer.

PS Carrier/Source	Exposure Context	Target Cell Type	Functional Consequence	Experimental Model	Key Reference
Viable tumor cell membrane	Hypoxia, metabolic stress, Ca^2+^ dysregulation	Macrophages, dendritic cells	Immune suppression, tolerogenic signaling	Human cancer cell lines; mouse tumor models	[15]
Viable tumor cell membrane	Chemotherapy/radiotherapy	T cells, NK cells	Resistance to immune-mediated killing	Mouse xenograft models	[16]
Tumor-associated endothelium	Inflammatory cytokines, ROS	Circulating immune cells	Vascular immune suppression, leukocyte exclusion	Mouse tumor vasculature	[14]
PS-rich extracellular vesicles (microvesicles, exosomes)	Tumor stress, apoptosis-independent shedding	Monocytes, macrophages	Systemic immune suppression	Patient plasma; mouse models	[33]
Apoptotic tumor cells	Caspase activation	Phagocytes	Efferocytosis, immune tolerance	In vitro apoptosis models	[3]
Circulating PS-positive vesicles	Tumor burden-dependent	Platelets, coagulation factors	Hypercoagulability, thrombosis	Patient plasma samples	[29]

Notes: PS, phosphatidylserine; ROS, reactive oxygen species. This table integrates cellular and vesicular PS exposure modes and emphasizes the experimental context in which each biological function was demonstrated.

**Table 3:** PS-Targeted Therapeutic Platforms: Mechanisms, Models, and Clinical Status.

Therapeutic Modality	PS-Targeting Mechanism	Disease/Model	Key Outcome	Development Stage	Key Reference
Bavituximab	Antibody binding to PS–β2GPI complex	Solid tumors	Immune reprogramming, vascular normalization	Phase II (completed)	[16]
SapC–DOPS (BXQ-350)	PS-binding lysosomal protein–liposome complex	Glioma, solid tumors	Tumor cell apoptosis, immune activation	Phase I/II	[35]
PS-targeted nanoparticles	Surface charge-mediated PS affinity	Multiple tumor models	Enhanced drug accumulation	Preclinical	[58]
PS-binding peptides	Electrostatic recognition of PS-rich membranes	Breast cancer, melanoma	Tumor targeting, drug delivery	Preclinical	[59]
PS-directed imaging agents	Annexin-V/PS-binding probes	Apoptosis, therapy response	Non-invasive PS visualization	Clinical imaging studies	[7]

Notes: β2GPI, beta-2 glycoprotein I. Therapeutic platforms are grouped by PS recognition strategy and annotated with experimental and clinical context.

## Data Availability

No new data were created or analyzed in this study. Data sharing is not applicable to this article.

## Supplementary Materials

The following supporting information can be downloaded at: https://www.mdpi.com/article/10.3390/ijms27020697/s1.

## Abbreviations

AC	Acylcarnitine
ADCC	Antibody-Dependent Cellular Cytotoxicity
ALS	Acidic Lysosomal Stress (or disease context dependent)
Arg-1	Arginase-1
ATP11A/C	P4-ATPase Aminophospholipid Flippases
Axl	TAM family receptor tyrosine kinase Axl
β2GP1	β2-Glycoprotein I
BXQ-350	Clinical-grade SapC-DOPS Nanovesicle
CAR-T	Chimeric Antigen Receptor T cell
CDC	Complement-Dependent Cytotoxicity
CTLA-4	Cytotoxic T-Lymphocyte Antigen 4
DC	Dendritic Cell
DOPS	Dioleoylphosphatidylserine
EC	Endothelial Cell
EV	Extracellular Vesicle (exosomes + microvesicles)
GBM	Glioblastoma Multiforme
GPCR	G-Protein Coupled Receptor
HIF	Hypoxia-Inducible Factor
HNSCC	Head and Neck Squamous Cell Carcinoma
IFN-γ	Interferon-gamma
IL-10	Interleukin-10
IL-12	Interleukin-12
LC-MS	Liquid Chromatography–Mass Spectrometry
LPS	Lipopolysaccharide
MerTK	Mer Tyrosine Kinase (TAM family)
MHC-II	Major Histocompatibility Complex Class II
NK cell	Natural Killer cell
NSCLC	Non-Small-Cell Lung Cancer
PD-1	Programmed cell death protein-1
PD-L1	Programmed death ligand-1
PDAC	Pancreatic Ductal Adenocarcinoma
PET/SPECT	Positron Emission Tomography/Single-Photon Emission CT
PS	Phosphatidylserine
ROS	Reactive Oxygen Species
SapC	Saposin C
TAM receptors	Tyro3, Axl, MerTK
TCR	T-Cell Receptor
TGF-β	Transforming Growth Factor-β
TIM-4	T-Cell/Transmembrane Immunoglobulin Mucin Domain-4
TMEM16F	TransMEMbrane protein family 16, member F; Ca^2+^-activated phospholipid scramblase (Anoctamin-6)
TME	Tumor Microenvironment
Treg	Regulatory T cell
VEGF	Vascular Endothelial Growth Factor
