# Supplementary material for: Phosphatidylserine Externalization in Cancer: Biology, Immune Suppression, and Emerging Theragnostic Strategies
Source: Int J Mol Sci. 2026 Jan 9;27(2):697. doi: 10.3390/ijms27020697 (PMC12841397; doi:10.3390/ijms27020697)
Supplement: Supplementary file 1 [file ijms-27-00697-s001.zip › ijms-4058242-supplementary.pdf]

Phosphatidylserine Externalization in Cancer: Biology, Immune Suppression, and Emerging Theragnostic Strategies

Supplementary Figure

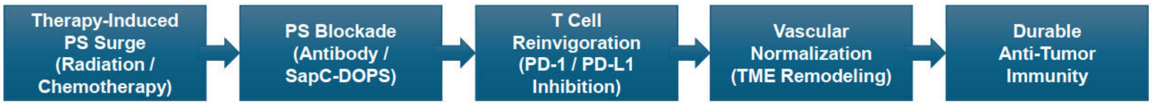

Supplementary Figure S1. Integrated PS-targeted therapeutic framework. Therapy-induced cellular stress—including radiation and DNA-damaging chemotherapies—triggers an acute surge of phosphatidylserine (PS) exposure on tumor cells and tumor-associated stromal compartments. This transient PS-high window provides an opportunity for PS-blocking agents (e.g., bavituximab, Annexin V–based probes, SapC–DOPS nanovesicles) to inhibit PS–TIM4/MerTK/Stabilin-2 signaling, suppress efferocytic and tolerogenic programs, and attenuate PS+ extracellular vesicle–mediated systemic immunosuppression. Subsequent PD-1/PD-L1 blockade promotes effector T-cell reinvigoration by restoring TCR signaling, reversing exhaustion, and enhancing intratumoral cytotoxicity. As PS-mediated endothelial dysfunction is alleviated, vascular normalization facilitates improved leukocyte trafficking and therapeutic penetration into the tumor microenvironment. Collectively, optimal sequencing of these steps supports the establishment of durable anti-tumor immunity, providing a mechanistic blueprint for rational PS-targeted combination therapies and their clinical translation.
